# Eyes with Suspicious Appearance of the Optic Disc and Normal Intraocular Pressure: Using Clinical and Epidemiological Characteristics to Differentiate Those with and without Glaucoma

**DOI:** 10.1371/journal.pone.0158983

**Published:** 2016-07-19

**Authors:** Diego T. Dias, Michele Ushida, Marina C. Sousa, Syril Dorairaj, Luis G. Biteli, Mauro T. Leite, Augusto Paranhos, Tiago S. Prata

**Affiliations:** 1 Department of Ophthalmology, Federal University of São Paulo, São Paulo, São Paulo, Brazil; 2 Glaucoma Unit, Hospital Medicina dos Olhos, Osasco, São Paulo, Brazil; 3 Department of Ophthalmology, Mayo Clinic, Jacksonville, Florida, United States of America; University of Houston, UNITED STATES

## Abstract

Among all glaucoma suspects, eyes with optic nerve head features suspicious or suggestive of early glaucoma are probably those that offer the greatest challenge for clinicians. In contrast with the robust longitudinal data published on ocular hypertension, there is no specific management guideline for these patients. Therefore, evaluating eyes with suspicious optic disc appearance and normal intraocular pressure (IOP), we sought to investigate potential differences in clinical and epidemiological characteristics to differentiate those with normal-tension glaucoma (NTG) from those with presumed large physiological optic disc cups (pLPC). In this observational case-control study, we consecutively enrolled individuals with pLPC and NTG. All eyes had vertical cup-to-disc ratio (VCDR)≥0.6 and untreated IOP<21 mmHg. Glaucomatous eyes had reproducible visual field defects. Eyes with pLPC required normal visual fields and ≥30 months of follow-up with no evidence of glaucomatous neuropathy. Clinical and epidemiological parameters were compared between groups. Eighty-four individuals with pLPC and 40 NTG patients were included. Regarding our main results, NTG patients were significantly older and with a higher prevalence of Japanese descendants (p<0.01). Not only did pLPC eyes have smaller mean VCDR, but also larger optic discs (p≤0.04). There were no significant differences for gender, central corneal thickness, and spherical equivalent (p≥0.38). Significant odds ratios (OR) were found for race (OR = 2.42; for Japanese ancestry), age (OR = 1.05), VCDR (OR = 5.03), and disc size (OR = 0.04; p≤0.04). In conclusion, in patients with suspicious optic disc and normal IOP, those with older age, Japanese ancestry, smaller optic discs, and larger VCDR are more likely to have NTG, and therefore, deserve deeper investigation and closer monitoring.

## Introduction

The term “glaucoma suspect”, advocated by Shaffer [1] in the 1970s, has been used to identify two main populations of individuals or eyes: those with consistently elevated intraocular pressure (IOP; ocular hypertensives) and those whose optic nerve head (ONH) and/or peripapillary retinal nerve fiber layer (pRNFL) appearance is suggestive of, but not definitive for, glaucoma [1–3]. Among all glaucoma suspects, eyes with optic nerve features suspicious or suggestive of early glaucoma are probably those that offer the greatest challenge for clinicians. In contrast with the robust longitudinal data published on ocular hypertension [4–8], there is no specific management guideline for patients with suspicious ONH appearance.

Identification of risk factors, including both ocular and systemic factors, associated with glaucoma development or disease progression has been the focus of numerous studies [4, 6, 8–16]. Known systemic risk factors for glaucoma progression include older age [6, 8–10], and lower systemic blood pressure [9]. For patients with normal-tension glaucoma (NTG), previously described factors include migraine and female gender [14]. Ocular risk factors reported to predict the development or progression of glaucoma include a thinner central corneal thickness (CCT) [6, 8, 9], worse visual field (VF) at baseline [6, 8, 9, 11], elevated IOP [4–13], increased cup-to-disc ratio [7, 8], and the occurrence of optic disc hemorrhage [15,16].

It is noteworthy that most studies investigating risk factors for glaucoma usually include a cohort of healthy individuals versus individuals with established glaucoma and/or elevated untreated IOP. However, on daily practice, we often deal with eyes with suspicious appearance of the optic disc (eg, those with large optic disc cups) and IOP within the normal range. In this scenario, at the time of diagnosis, it is not an easy task to determine whether a patient has NTG or just a large physiological optic disc cup (pLPC). The knowledge of factors associated with the presence of NTG in this specific population would help clinicians to determine patient follow-up and diagnosis. In the present study, evaluating eyes with suspicious appearance of the optic disc and normal IOP, we investigated potential differences in clinical and epidemiological characteristics to differentiate those with NTG from those with pLPC.

## Materials and Methods

### Ethics Statement

This prospective protocol adhered to the tenets of the declaration of Helsinki and was approved by the Ethics Committee of the Federal University of São Paulo. In addition, written informed consent was obtained from all participants.

### Participants

In this observational case-control study, participants were consecutively recruited from the Federal University of Sao Paulo (Sao Paulo, Brazil) and from Hospital Medicina dos Olhos (Osasco, Brazil) between June 2012 and June 2013. All participants underwent a comprehensive ophthalmological evaluation, including best-corrected visual acuity, slit-lamp biomicroscopy, IOP measurement, gonioscopy, dilated fundoscopy, VF testing (SITA), and optic disc stereophotographs.

To be included, eyes with pLPC required normal VF testing and suspicious appearance of the optic disc (defined as a vertical cup-to-disc ratio [VCDR]≥0.6), but without any definitive sign of glaucoma (eg, localized pRNFL defects and/or neuroretinal rim defects) and had at least 30 months of follow-up with no evidence of progressive optic neuropathy (assessed by serial color stereophotographs). Based on the ISGEO classification, in most studies the VCDR cut-off value used to separate glaucomatous from healthy eyes was usually determined as 0.7 (based on the 97.5 percentile of the VCDR distribution for the studied population). In the present study, our goal was to include glaucomatous and suspect eyes, not healthy eyes. Therefore, we adopted a less strict cut-off value (≥0.6), which we considered more clinically relevant, as many eyes with a VCDR of 0.6 would be probably classified as suspects on daily practice [17, 18]. Also, they were required to have IOP<21mmHg during the follow-up period and no previous history of IOP-lowering medications. Eyes with NTG had to have untreated IOP<21 mmHg, evidence of glaucomatous optic neuropathy and reproducible glaucomatous VF defects. Exclusion criteria for both groups were gonioscopically occludable angles (defined as an eye in which the posterior trabecular meshwork was not visible for ≥180° on dark-room gonioscopy), previous ocular surgery or trauma, use of oral or topical steroids, and ocular diseases other than glaucoma. Two experienced graders evaluated all stereophotographs (masked to patient’s clinical data); in case of disagreement a third grader was used to adjudicate.

Characteristic glaucomatous optic neuropathy was defined as a vertical VCDR≥0.6, asymmetry of VCDR≥0.2 between eyes, presence of localized pRNFL defects and/or neuroretinal rim defects in the absence of any other abnormalities that could explain such findings. A glaucomatous VF defect in the standard automated perimetry (Humphrey SITA—Standard 24–2, Carl Zeiss Meditec, Dublin, CA) was defined as three or more points in clusters with a probability of <5% (excluding those on the edge of the field or directly above and below the blind spot) on the pattern deviation plot, a pattern standard deviation index with a probability of <5%, or a glaucoma hemifield test with results outside the normal limits.

### Data Collection and Statistical analysis

The following demographic and ocular characteristics were collected and compared between the groups: age, gender, race, CCT (ultrasound pachymetry), refractive error (spherical equivalent), and optic disc characteristics (optic disc area and VCDR). Optic disc parameters were based on spectral-domain optical coherence tomography (SD-OCT) measurements obtained with the RTVue-100 OCT (Optovue, Inc., Fremont, CA; software version A4).

Descriptive statistics included mean and standard deviation for normally distributed variables and median, quartiles for non-normally distributed variables. Continuous variables with normal distribution were compared using independent samples t-test while those non-normally distributed were analyzed using Mann-Whitney test. Categorical data were analyzed using chi-square test. Multiple logistic regression analysis was used to identify possible factors (and respective odds ratios [OR]) associated with the presence of glaucoma in these patients. First, each variable was analyzed in a univariable model. Then, all variables with a significance level (*P*) of less than 0.10 were included in the multivariable model. All statistical analyses were performed using Stata (Stata version 10; StataCorp, College Station, TX). The alpha level (type I error) was set at 0.05.

## Results

A total of 84 eyes with pLPC (84 patients) and 40 NTG eyes (40 patients) were included (S1 File). Table 1 provides clinical and demographic characteristics of included patients. NTG patients were significantly older (57.1±13.9 vs 46.5±15.3 years; p<0.01) with a higher prevalence of Japanese descendants (p<0.01) when compared to patients with LPC. Not only did pLPC eyes have a smaller mean VCDR compared to NTG eyes (0.66 vs 0.73; p<0.01), but also larger optic disc sizes (median of 2.47 vs 2.16 mm^2^; p = 0.04). In fact, 90% of the individuals with pLPC presented with a maximum VCDR of 0.75. We found no significant differences in gender, CCT, and spherical equivalent between groups (p≥0.38). Multivariate logistic regression analysis revealed significant OR for race (OR = 2.42; p = 0.03; for Japanese ancestry), age (OR = 1.05 p = 0.04; for each year), VCDR (OR = 5.03; p<0.01; for each 0.1 in VCDR), and disc size (OR = 0.04; p<0.01; for each 1mm^2^).

**Table 1:** Demographic and ocular characteristics of individuals with presumed large physiological cupping and normal-tension glaucoma patients.

Parameters*	Presumed Large Physiological Cupping (n = 84 patients)	Normal-Tension Glaucoma (n = 40 patients)	P value
Age (years)	46.5±15.3	57.1±13.9	<0.01
Sex (% female)	62%	68%	0.65
Race (W/AD/JD/O)	69% / 13% / 8% / 10%	45% / 10% / 32% / 13%	<0.01
CCT **(**μm)	536.1±35.6	529.5±31.9	0.38
Spherical Equivalent (D)	0 (-1.25, 1.00)	0 (-1.31, 1.12)	0.93
VCDR	0.66±0.1	0.73±0.1	<0.01
Optic disc area (mm^2^)	2.47 (2.12, 2.82)	2.16 (1.93, 2.54)	0.04

W, White; AD, African descent; JD, Japanese descent; O, Others; CCT, central corneal thickness; D, diopters; VCDR, vertical cup-to-disc ratio.

*Normally distributed variables represented by mean±standard deviation; non-normally distributed variables represented by median (first quartile, third quartile).

## Discussion

Although a common scenario on a clinical setting, there is scant information in the literature or guidelines about how we should manage patients with suspicious appearance of the optic disc and IOP within the normal range. Unless a patient presents with anatomical (eg, localized pRNFL or neuroretinal rim defects) or functional findings strongly suggestive of glaucomatous neuropathy, he or she is usually followed as a suspect indefinitely. In addition, although the natural history of the disease in untreated glaucomatous patients with normal IOP varies significantly, changes are typically small and slow, often insufficient to measurably affect VF indices even after five years of follow-up [19]. It’s noteworthy that SD-OCT also has a limited diagnostic ability to differentiate between patients with pLPC and glaucoma with IOP within the normal range [20]. Therefore, the knowledge of factors associated with the presence (or absence) of glaucoma in patients with suspicious optic discs would likely improve our ability to manage these cases, especially during the first initial visits, when usually there are no previous follow-up data (VFs and disc photographs) available. Using a case-control study design, we identified factors associated with the presence of NTG in this specific population. The authors are unaware of any previously published study with a similar purpose and design.

The vast majority of previous studies evaluating factors associated with NTG have focused on risk factors for disease progression, not development. In this context, the most robust data probably comes from the Collaborative Normal-Tension Glaucoma Study [14]. The main purpose of the study was to uncover risk factors for the highly variable individual rates of progression in cases of untreated NTG. In sum, female gender, migraine, and the occurrence of optic disc hemorrhages were associated with faster VF deterioration over time. More recently, studies have underscored the role of low ocular perfusion pressure and greater extent of myopia as significant risk factors for disease progression in these patients, with a preferential deterioration of the central and paracentral VF areas [21–23]. Although all these findings have a great clinical importance as they influence the prognosis of the individual's disease and thereby the frequency of follow-up and aggressiveness of the therapy to be undertaken, they exclusively represent risk factors for disease progression and are not directly related to the risk of having NTG or not once you have a suspicious optic disc.

When it comes to studies assessing patients’ clinical and epidemiological characteristics as possible factors to differentiate pLPC from glaucomatous cupping, there are not much data available. A recent study by Lee et al compared the characteristics of patients with a localized RNFL defect and normal optic disc appearance (unchanged for more than five years) with those from patients with NTG [24]. A total of 40 patients were included in each group and the main variable evaluated was the angle of each RNFL defect. The authors found that NTG is less likely in eyes with suspicious optic disc appearance if a patient has systemic diseases and the distal borders of the RNFL defects are closer to the macula. In our study, we identified different factors associated with the presence of NTG, but systemic diseases and the angles of RNFL defects were not investigated. In fact, all variables were chosen based on previous publications of risk factors for glaucoma development and/or progression [4, 6, 8–16], and on the initial comparison performed between the two selected populations in our study (Table 1). In addition, inclusion criteria for the control groups differed significantly between the present study and that from Lee et al [24]. Therefore, we believe that results from these two studies should not be directly compared. On the other hand, they seem to add significantly to each other.

At this point, we believe it is important to discuss the clinical meaning and rationale of our findings.

### Older age

The association between age and glaucoma prevalence has been underscored in several studies; older patients having a greater risk of developing the disease [8–10, 14, 25]. Corroborating these findings, biomechanical studies have proposed that age-related alterations in the optic nerve head underlie the clinical behavior and increased susceptibility of the aged ONH to glaucomatous damage [26]. Based on our results, this relationship between age and glaucoma also seems to be valid for patients with suspicious optic disc and IOP within the normal range.

### Japanese ancestry

NTG is reported to be the most common form of glaucoma in Japan [27]. Although all patients from our study were Brazilians, 32% of those with NTG were Japanese descendants (in contrast with only 8% of those without glaucoma). When it comes to race composition, the Brazilian population is very heterogeneous, with a predominance of white people in the region where the study was conducted. We believe that the relationship we uncovered between Japanese ancestry and the presence of glaucoma in these patients with suspicious optic disc appearance and normal IOP is probably explained by the fact that Japanese descendants in Brazil are also more likely to have NTG than patients from other races.

### Optic disc size and cup-to-disc ratio

In healthy individuals, it is well known that larger cups are more often observed in eyes with large optic discs, while smaller cups are found in those with small discs. Data regarding patients with pLPC report an increase of 0.21 in VCDR for each 1mm^2^ in disc area [20]. Regarding cup size, the presence of a larger VCDR has been shown to be a significant independent predictor of disease onset over time in glaucoma suspects (ocular hypertensives) [6, 8]. Even though we evaluated a different population (eyes with suspicious appearance of the optic disc with normal IOP), our findings indirectly corroborate these previously reported data, as we identified that eyes with a smaller VCDR and larger optic discs are more likely to have solely a large physiological cupping, and not NTG.

The present study has some specific characteristics and limitations that should be addressed. First, we defined IOP within the normal range without considering diurnal IOP variation and CCT influence on applanation tonometry measurements. Second, it is possible that some eyes with pLPC will develop glaucomatous progression over time. By including pLPC eyes with at least 30 months of follow-up without progression, we expect to reduce this occurrence. Third, other systemic and ocular factors that could be potentially associated with the presence of NTG in this population, such as migraine, axial length and corneal hysteresis, were not evaluated in the present study. Finally, although the case-control design of the study allows the investigation of possible associations, it does not provide cause-effect relationships. A longitudinal analysis following solely pLPC eyes over time is warranted in order to establish risk factors for conversion to glaucoma. These limitations should be considered while interpreting our results.

In conclusion, while dealing with patients with suspicious appearance of the optic disc and normal IOP (a common and challenging situation on daily practice), we identified independent clinical and ocular characteristics that might help clinicians to distinguish between those with and without NTG. Patients with older age, Japanese ancestry, smaller optic discs and larger vertical cup-to-disc ratio are more likely to have NTG and not just a large physiological cupping, and therefore deserve deeper investigation and closer monitoring.

## Supporting Information

S1 File Raw demographic and ocular characteristics of individuals with presumed large physiological cupping (pLPC) and normal-tension glaucoma (NTG) patients. This file presents all raw demographic and ocular data of the 84 pLPC and the 40 NTG eyes included in this study.
(XLSX)
